# 3D-Printed masks as a new approach for immobilization in radiotherapy – a study of positioning accuracy

**DOI:** 10.18632/oncotarget.24032

**Published:** 2018-01-08

**Authors:** Matthias Felix Haefner, Frederik Lars Giesel, Matthias Mattke, Daniel Rath, Moritz Wade, Jacob Kuypers, Alan Preuss, Hans-Ulrich Kauczor, Jens-Peter Schenk, Juergen Debus, Florian Sterzing, Roland Unterhinninghofen

**Affiliations:** ^1^ Department of Radiation Oncology, Heidelberg University Hospital, 69120 Heidelberg, Germany; ^2^ National Center for Radiation Research in Oncology (NCRO), Heidelberg Institute for Radiation Oncology (HIRO), 69120 Heidelberg, Germany; ^3^ Department of Nuclear Medicine, Heidelberg University Hospital, 69120 Heidelberg, Germany; ^4^ Institute of Antropomatics and Robotics, Karlsruhe Institute of Technology (KIT), 76131 Karlsruhe, Germany; ^5^ Department of Diagnostic and Interventional Radiology, Heidelberg University Hospital, 69120 Heidelberg, Germany; ^6^ Department of Radiation Oncology Kempten, 87439 Kempten, Germany; ^7^ Institute of Robotics and Mechatronics, German Aerospace Center, 82234 Oberpfaffenhofen-Weßling, Germany

**Keywords:** immobilization, radiotherapy, 3D-printing, setup accuracy, head mask

## Abstract

We developed a new approach to produce individual immobilization devices for the head based on MRI data and 3D printing technologies. The purpose of this study was to determine positioning accuracy with healthy volunteers.

3D MRI data of the head were acquired for 8 volunteers. In-house developed software processed the image data to generate a surface mesh model of the immobilization mask. After adding an interface for the couch, the fixation setup was materialized using a 3D printer with acrylonitrile butadiene styrene (ABS). Repeated MRI datasets (n=10) were acquired for all volunteers wearing their masks thus simulating a setup for multiple fractions. Using automatic image-to-image registration, displacements of the head were calculated relative to the first dataset (6 degrees of freedom).

The production process has been described in detail. The absolute lateral (x), vertical (y) and longitudinal (z) translations ranged between −0.7 and 0.5 mm, −1.8 and 1.4 mm, and −1.6 and 2.4 mm, respectively. The absolute rotations for pitch (x), yaw (y) and roll (z) ranged between −0.9 and 0.8°, −0.5 and 1.1°, and −0.6 and 0.8°, respectively. The mean 3D displacement was 0.9 mm with a standard deviation (SD) of the systematic and random error of 0.2 mm and 0.5 mm, respectively.

In conclusion, an almost entirely automated production process of 3D printed immobilization masks for the head derived from MRI data was established. A high level of setup accuracy was demonstrated in a volunteer cohort. Future research will have to focus on workflow optimization and clinical evaluation.

## INTRODUCTION

In conformal radiotherapy, accurate patient immobilization is crucial to guarantee an optimal dose coverage of the target volume while healthy tissue is not affected unduly. This applies particularly to the head and neck area where tumors typically reside in the immediate vicinity of organs at risk such as the brain stem or the spinal cord and safety margins are accordingly narrow [1]. In practice, common guidelines have been established to manage margins and to minimize errors [2]. However, as irradiation technology advances allowing for steeper gradients and more exact dose planning and delivery resulting in a higher sensitivity to setup uncertainties [3, 4], patient immobilization needs to keep pace.

Today, immobilization of the head is most commonly accomplished by thermoplastic masks [5]. Occasionally, the masks are combined with bite blocks in order to enhance the immobilization effect. Alternative, though more rarely used immobilization devices include Scotchcast masks, bite blocks alone and stereotactic frames using invasive screwing.

The most important attribute of an immobilization device is its intrafractional and interfractional accuracy describing how far a patient can move her or his head while wearing a mask and how well the positioning of the patient is reproducible for multiple fractions, respectively. For both aspects, mean uncertainties of < 5 mm are described [6–12].

Regardless of the respective means, immobilizing the head is more delicate than it is with other parts of the body. Besides the general incidence of anxiety prior to radiotherapy [13] mask fixation of the head causes additional mental stress, physical discomfort up to pain or even claustrophobia in a considerable number of patients [14–16]. Hence, there is an obvious need to improve wearing comfort of immobilization masks as well as to ease the production process when patients usually get in touch with the mask for the first time. Furthermore, while many processes in radiation oncology have undergone dramatic changes in terms of computer based automation and calculation, the manufacturing of immobilization devices is still a manual process requiring human resources, room, materials and storage.

The technique of 3D-printing, also referred to as rapid prototyping, has commenced a revolution in several industries owing to the fast and increasingly cost-effective production of individual 3D objects from digital models [17]. In the past two decades, 3D-printing has also been implemented for medical purposes, e.g. for surgical planning, implant design or research and training models [18]. In the field of radiotherapy, rapid prototyping is used rather sporadically, but has mainly been introduced to create custom devices for beam range modulation [19, 20], dosimetry [21, 22] or brachytherapy application [23, 24].

Rapid prototyping has huge potential to create individual and customizable immobilization devices that overcome or at least improve most of the previously mentioned disadvantages of currently used fixation systems. In a proof of principle article, Sanghera et al. described the basic production process of a rudimentary 3D-printed facemask derived from optical surface scanning data [25].

Our group developed a new approach to produce head immobilization devices with rapid prototyping based on magnetic resonance imaging (MRI) data. In this article, we introduce the production process including the establishment of a feasible workflow and present the results of a volunteer-based study investigating the setup accuracy of the system.

## RESULTS

Mask models were computed successfully for all volunteers using the automated processing software. Only little manual intervention was required in some cases for adjusting the position of the apertures for eyes, nose and mouth. The printing process of the masks was performed without any complications.

From a first subjective impression during the initial try-on, all masks fitted tightly to the volunteers’ faces hardly allowing any motion. The masks were concordantly perceived comfortable without any painful squeezing or pinching in the head and neck area. As essential part of the immobilization device not only the mask, but also the uni-size headrest fitted all volunteers and was assessed convenient.

### Positioning accuracy

**Figure 1:**
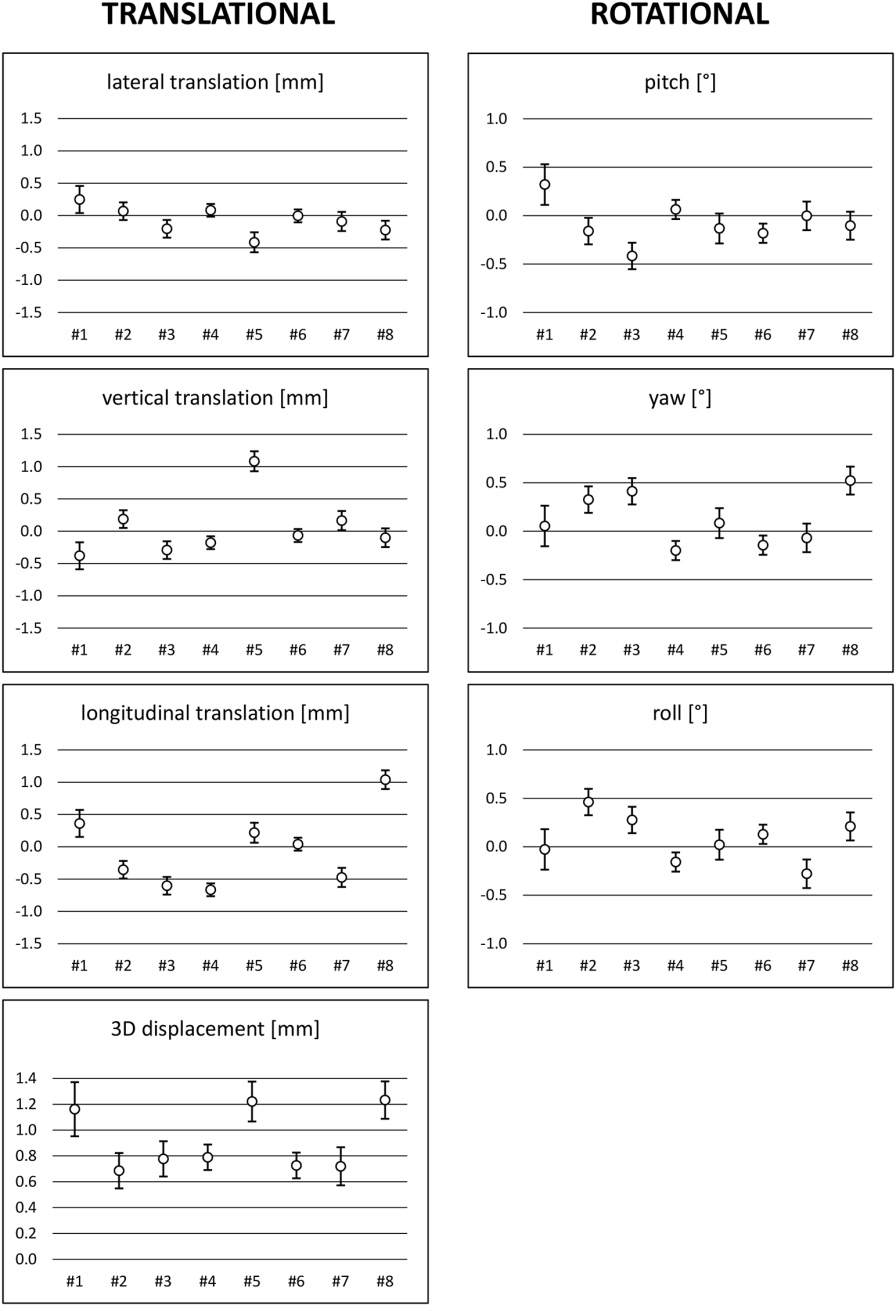
Plots of individual translational and rotational errors for volunteers #1 to #8 (mean and standard deviation)

Eight healthy volunteers (n=4 female, n=4 male) aged between 20-32 years took part in the study. For ten simulated radiotherapy fractions, the absolute lateral (x), vertical (y) and longitudinal (z) translations ranged between −0.7 and 0.5 mm, −1.8 and 1.4 mm, and −1.6 and 2.4 mm, respectively. Concerning rotational movement, the minimum and maximum registered angles were −0.9 and 0.8° for pitch (x), −0.5 and 1.1° for yaw (y), and −0.6 and 0.8° for roll (z). Individual translational and rotational values for all volunteers are shown in Figure 1. The mean 3D displacement was 0.9 mm with a standard deviation (SD) of the systematic error of 0.2 mm and a SD of the random error of 0.5 mm. Mean values as well as SD of systematic and random errors are separately shown in Table 1 for all six degrees of freedom.

**Table 1:** Mean group translational displacement and rotational movement with standard deviations (SD) of systematic and random errors

Errors		Group mean	SD of systematic error	SD of random error
Translations				
lateral (x)	[mm]	-0.1	0.21	0.14
vertical (y)	[mm]	0.1	0.46	0.29
longitudinal (z)	[mm]	-0.1	0.58	0.71

3D displacement	[mm]	0.9	0.24	0.46
Rotations				
pitch (x)	[°]	-0.1	0.21	0.29
yaw (y)	[°]	0.1	0.27	0.24
roll (z)	[°]	0.1	0.24	0.22

## DISCUSSION

With the presented study, we are able to demonstrate practical feasibility of 3D-printed masks as head immobilization devices for radiotherapy by developing the basic concept and parts of the software, designing all components including the headrest and the mask itself and establishing a production workflow. Moreover, we could validate the functionality of the system by proving excellent setup accuracy which is the central benchmark for immobilization systems.

The basic idea of creating facemasks for radiotherapy from digitally processed surface data was first described by Sanghera et al. in 2002 in a proof of principle paper using an optical surface scanner [25]. Simultaneously to our efforts a group of UK researchers has developed an algorithm to create facemasks derived from DICOM-datasets and investigated the basic impact on radiation dose [26]. They were able to demonstrate preclinical feasibility in principle and comparable dose interference of certain rapid prototyping materials compared to a standard thermoplastic mask. With our presented results, we take that approach to a significantly higher level of development as we took account of the wearing comfort, an interface solution to imaging and radiation treatment devices and the setup accuracy. Thus, we provide a solution that is ready to use in a clinical setting. For example, pretreatment diagnostic MRI imaging may be performed using the 3D-printed headrest to serve as image source for the 3D-model. After fabrication, the patient is able to undergo her or his planning CT wearing the printed mask, thus entering the usual radiation treatment planning workflow.

Accurate and reliable immobilization is crucial for high quality radiotherapy. For intracranial stereotactic radiosurgery (SRS), Guckenberger et al. were able to show that target coverage and dose conformity are reduced to 75% (range 56-94%) and 60% (range 35-85%), respectively, when waiving image guidance and that each setup error of 1 mm results in a decrease of coverage and conformity by 6% and 10% on average, respectively [10]. Similar observations on the negative influence of setup errors to treatment quality have been reported for normofractionated intensity-modulated radiotherapy (IMRT) in head and neck cancer patients [4, 27].

The setup accuracy of a mask immobilization system is mainly determined by its material and its design. For most commonly used thermoplastic masks, it ranges between 2 and 5 mm [6, 8-10, 12, 28, 29]. A separate fixation of the shoulders can reduce uncertainties at that level [6], but do not impact positioning accuracy of intracranial radiotherapy targets [9]. The best results for thermoplastic based devices were achieved by combination of the mask with a mouthpiece resulting in a mean translational error of 2.1 mm [9, 29] and a potential reduction of rotational errors [29]. In our study, the mean overall displacement vector of the system was 1.3 mm (SD 0.53 mm) even without mouthpiece. Thus, the 3D-printed mask ranks on a level with the best performances reported for clinically applied thermoplastic systems. It may even measure up to more rigid mask materials, e.g. made of scotchcast, that provide mean setup errors of less than 2 mm as shown by Karger et al. [30] and show slight positioning superiority in direct comparison to thermoplastic systems [8].

Mean intrafractional shifting mostly ranges in the submillimeter area or < 2 mm at any rate and increases with the duration of immobilization for SRS-dedicated thermoplastic or scotchcast masks [8–12, 31]. Studies investigating both displacement components consistently report on lower intrafractional than interfractional shifting vectors for the same immobilization device [8-10, 12]. Thus, we have reason to assume that our system results in a comparable intrafractional positioning accuracy although we did not explicitly evaluate it in this study.

It is obvious that both interfractional and intrafractional accuracy are substantially determined by the actual movement of the subject or patient in the mask. However, other aspects of immobilization and imaging influence the size and direction of the displacement vectors measured. By using six degrees of freedom (DOF) correction vectors are considerably reduced compared to a 3-DOF-based registration [31, 32]. An additional impact on accuracy is reported in terms of modality and quality of image-guidance [33]. And finally, from a practical point of view, displacement vectors may differ for the same person in the same immobilization device depending on the reference point of registration. For mask systems, this is of great importance when it comes to the treatment of larger target volumes in the head and neck area as different studies have revealed increasing positioning uncertainties in the lower parts of the neck [6, 8, 28, 34].

Especially for treatment sites located in the head and neck area the influence of immobilization devices on dosimetry has to be taken into account. Whereas beam attenuation is automatically accounted for in CT-based treatment planning and thus rather negligible the potential bolus effect of mask material increasing surface or skin dose, respectively, has further implications. Using thermoluminescence dosimeters (TLD) Lee et al. have shown an increase of surface dose up to 18% for 3 mm thick thermoplastic material [35]. Of greater importance, it has been shown that immobilization masks may lead to increased skin toxicity in head and neck cancer patients [4, 12]. Though dosimetric specifications of the material used in this study (ABS) are not available yet, we expect characteristics comparable to other rapid prototyping or thermoplastic materials based on the findings by Laycock et al [26]. Furthermore, the mask system presented in this study might be able to limit skin toxicity due to a thickness of only 1.5 mm and its potential to create additional, customized cutouts.

There are some limitations of our study. Firstly, subjects are not patients and a study environment does not reflect daily routine in radiation oncology. Hence, only clinical use will allow to draw conclusions on practical aspects like handling or patient comfort. Secondly, important factors like cost and time effectiveness can currently not compete with standard systems in use. However, as our study was to demonstrate the prototype development and first practical validation, its scope was consequently not to present a product ready for the market that has to compete with standard systems in terms of economic viability. Considering the already high degree of automating in the fabrication process and the significant and continuing reduction of production costs for rapid prototyping the presented approach has the potential to serve as reasonable alternative to established immobilization devices in the future. Before that, of course, several aspects like a speed-up of production time or an extension of suitable volumetric source data have to be advanced.

The presented head immobilization system has several strengths. Firstly and most importantly, setup accuracy was excellent and in line with standard systems in practical use. Secondly, the high degree of automating provides the groundwork for scalability for larger patient numbers and the adaptability of the system offers multiple options of customization including the development of immobilization devices for other parts of the body, e.g. extremities. Thirdly, the patient does not have to undergo the potentially discomforting procedure of shaping the mask on her or his face. As an alternative to existing, more or less practicable suggestions [36, 37] individual mask customization may also help reducing anxiety and improving comfort in claustrophobic patients.

In conclusion, based on medical imaging data rapid prototyping provides accurately fitting immobilization masks for the head. The almost completely automated production process is contact-free.. Setup accuracy is excellent and keeps up with superior immobilization systems established in modern radiotherapy. Future efforts have to focus on practical usability of the mask system, advance procedural methods to increase temporal and financial effectiveness and investigate the potential of individual customization.

## MATERIALS AND METHODS

### Mask design and development

**Figure 2:**
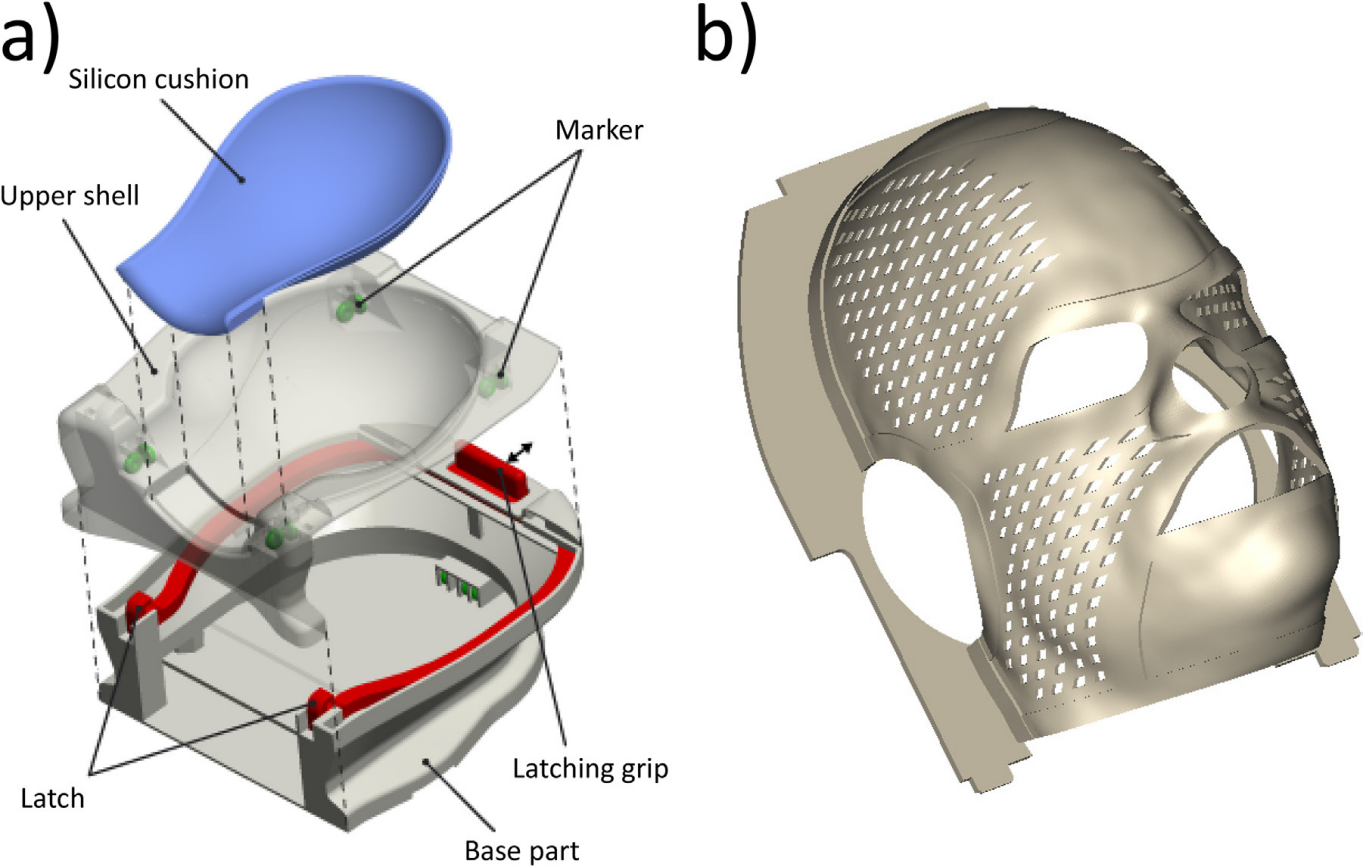
Production process of an individual immobilization mask

Our system of 3D-printed head masks consists of two parts: a headrest and the mask itself (Figure 2). While the mask is produced individually for each patient based on image data, the headrest is standardized and reusable. In principle, a range of headrests for small, mid-size and large heads may be provided.

The headrest was designed using commercial computer-aided design (CAD) software (Creo Parametric 2.0, PTC, Needham, MA, USA) and printed from acrylonitrile butadiene styrene (ABS) plastic in fused deposition modeling (FDM) technique using a Stratasys Dimension SST1200es 3D printer (Stratasys, Eden Prairie, MN, USA). It is basically a half shell for the back of the head with an integrated soft silicon cushion (hardness: shore A 20) for increased comfort. The surrounding frame provides a latching mechanism to fasten the mask. On the bottom side, special bores allow for connection to the treatment table using indexing bars.

**Figure 3:**
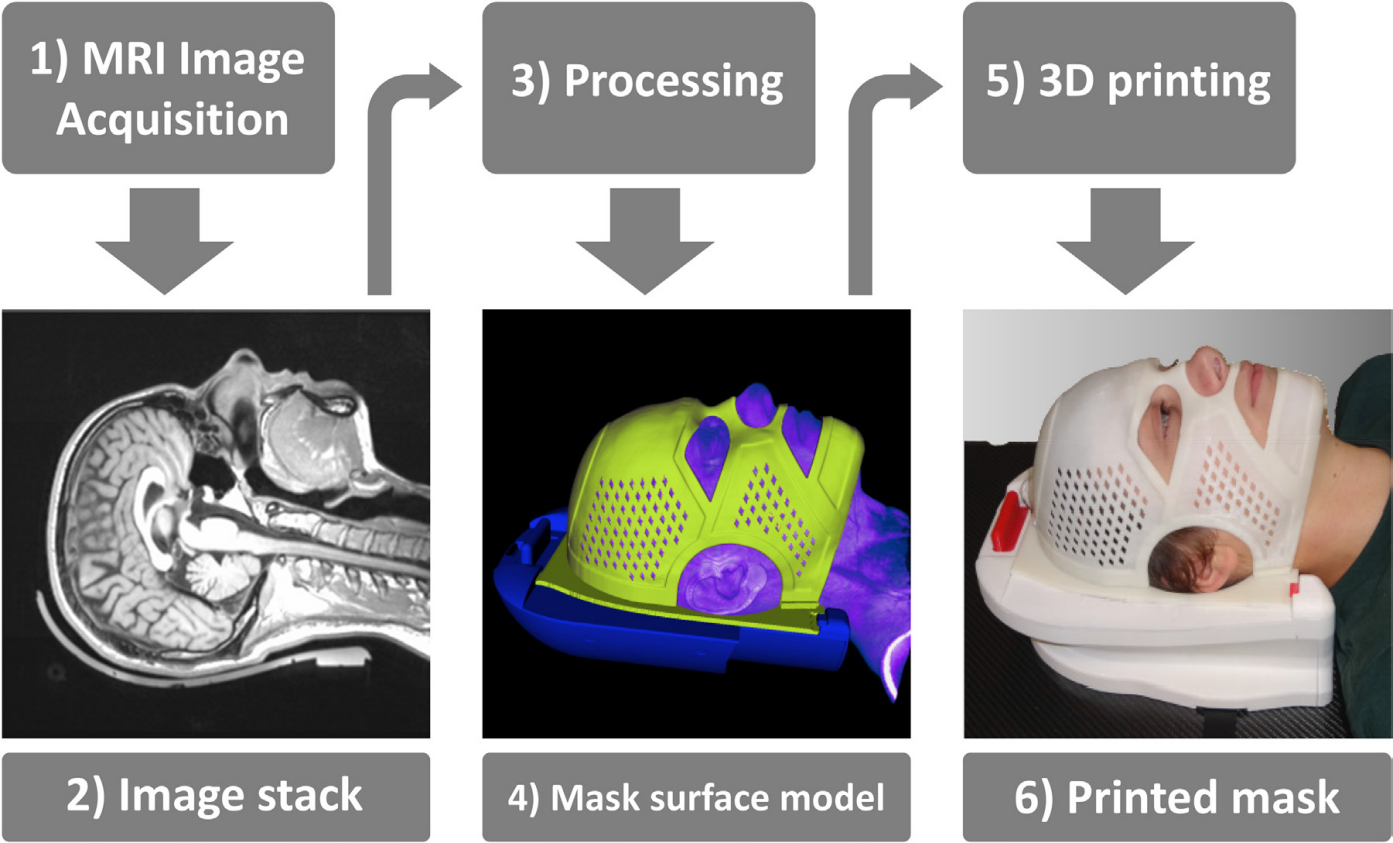
Flow chart of the study sequence

Dedicated software was developed in-house to generate individual mask models from image data that are then materialized using the 3D printer as specified above (Figure 3). During imaging the patient lies on the headrest. The software automatically segments the head surface from MRI data and detects specific markers integrated into the headrest to ensure proper registration of the mask with the latching mechanism. Subsequently, the head surface is further processed until it is the production-ready mask model. The mask has a thickness of 1.5 mm and features apertures for eyes, ears, nose and mouth. Also, a number of small holes is provided in order to reduce perspiration and to give the mask a friendlier appearance. To enhance stability ABS was reinforced by +1 mm around the apertures and the outer parts of the mask itself. At the back side of the mask a frame builds the interface to the headrest.

### Study setup

**Figure 4:**
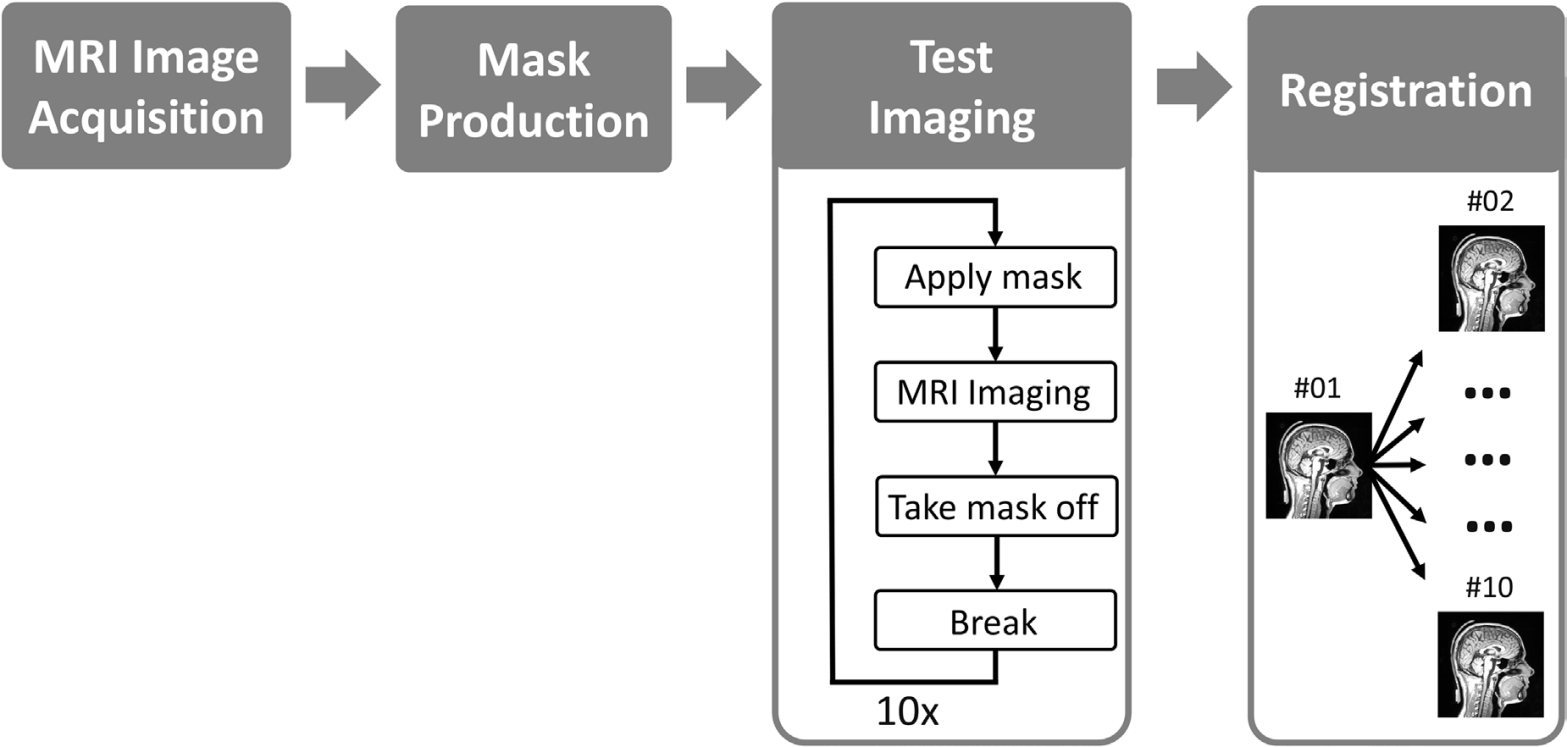
a) Structure of the headrest and b) 3D-model of a face mask

The study was approved by the institutional ethical review committee and all participating volunteers gave written informed consent prior to the beginning of the study. Eight healthy volunteers were prospectively evaluated. The study sequence is outlined in Figure 4 and basically contains four phases: (1) baseline imaging, (2) mask production, (3) test imaging and (4) evaluation. Each phase is described separately in the following subsections.

### Baseline imaging

Initial imaging was performed using a Magnetom Avanto 1.5T MRI system (Siemens, Erlangen, Germany) equipped with a 12-element head matrix coil and 4-element neck matrix coil combination. Since the headrest described above is too large to fit into the head coil, a dedicated version of the headrest was designed to exactly fit into the lower coil part. Its interfacing is compatible however, so that masks produced from MRI data may be used with the normal headrest and vice versa.

All volunteers were in supine position with their heads placed on the headrest and the upper parts of the head and neck coils put in place. Nose position was centered and pointed upright. The volunteers were asked to avoid any movement during image acquisition. A single 3D sagittal image stack of the head was acquired within 6 minutes using a standard T1-weighted magnetization-prepared rapid gradient echo sequence (MPRAGE). Matrix size was 248 x 256 x 192 mm^3^ and voxel size was 1.25 x 1.25 x 1.00 mm^3^.

From pilot testing the imaging modality was known to slightly suffer from insufficient dimensional accuracy. Distances between the markers in the headrest as determined from the images had turned out to deviate from the actual distances. The problem was solved by performing 3D distortion correction using the algorithm provided by the Syngo software (Version B17, Siemens, Erlangen, Germany).

### Mask production

One individual mask was produced for each volunteer following the workflow described above. Computation and fabrication of the masks didn’t cause any difficulties.

### Test imaging

Imaging setup and technical parameters were identical to the baseline examinations including the use of 3D distortion correction. We performed test imaging sessions with two volunteers at a time who were alternately positioned in the MRI scanner wearing their individual mask up to a total of ten imaging sets per volunteer. Hence, after a scanning phase each volunteer had to take off her or his mask and put it back on for the next one. Therefore, the study investigates repositioning accuracy which translates to interfractional reproducibility in a clinical context with patients. Accordingly, for a total of 8 volunteers a total number of 80 MRI data sets was generated.

### Evaluation

To evaluate repositioning accuracy of the masks, control image sets #2 through #10 were compared by pairs to set #1, and the respective displacements of the head were computed using an automated image processing pipeline. Rigid image registration was implemented with Insight Toolkit (ITK, www.itk.org). The GradientDescentLineSearch algorithm was used to perform transformation in six degrees of freedom (DOF), i.e. three translations as vectors and three rotations as versors. Technically, two consecutive registrations were performed for each set of pairs with #1 as reference and #2 to #10 as moving images. In the first step, the moving images were registered using the headrest markers. Subsequently, the head was isolated in all images, i.e. all markers and other visible parts of the headrest were removed, and it was transformed into the coordinates of the reference image set. In the second step, the isolated head images were registered using brain structures for image-to-image registration. The registration result was critically reviewed by an expert including the assessment of quality criteria that were automatically provided by the software and manually corrected, if necessary. For each subject and registration process, translational shifts (x: lateral/mediolateral, y: vertical/anterior-posterior, z: longitudinal/cranio-caudal), rotational shifts (x: pitch, y: yaw, z: roll) and the sum of all three translational shifts (3D displacement = √x^2^+y^2^+z^2^) were tabulated and resulting mean values and standard deviations (SD) were calculated. For the complete group of subjects, the mean error as well as the SD of systematic and random errors were determined for all shifts.
